# Konditionierung mit Ganzkörperbestrahlung oder Chemotherapie bei ALL im Kindesalter: Eine multinationale, randomisierte Phase-III-Studie auf Nichtunterlegenheit

**DOI:** 10.1007/s00066-021-01805-1

**Published:** 2021-07-02

**Authors:** Martin G. Sauer

**Affiliations:** Bereich Pädiatrische Stammzelltransplantation und Zelltherapie, Klinik für Pädiatrische Hämatologie und Onkologie, Medizinische Hochschule Hannover, Carl-Neuberg-Straße 1, OE 6780 Hannover, Deutschland

## Fragestellung

Die Ganzkörperbestrahlung (TBI) als Bestandteil der Konditionierung vor allogener Blutstammzelltransplantation (HSCT) ist effektiv bei Kindern mit Hochrisiko-ALL. Die Spätfolgen der Bestrahlung können allerdings substanziell sein. Deswegen wurde die Frage gestellt, ob eine reine Chemokonditionierung die Ganzkörperbestrahlung ersetzen kann.

## Patienten und Methoden

Die Studie (*Akronym:* FORUM) wurde als randomisierte, kontrollierte, internationale open-label Phase-III-Studie auf Nichtunterlegenheit konzipiert und multizentrisch durchgeführt. Kinder und Jugendliche, die sich zum Zeitpunkt der HSCT in kompletter Remission befanden, 4–21 Jahre alt waren und einen HLA-kompatiblen Spender hatten, wurden zwischen einer Konditionierung mit 12 Gy TBI und Etoposid oder einer reinen Chemokonditionierung mit Fludarabin, Thiotepa und entweder Treosulfan oder Busulfan randomisiert. Die Schwelle für Nichtunterlegenheit wurde auf 8 % festgesetzt. Bei 1000 zu randomisierenden Patienten innerhalb von 5 Jahren, einer Nachbeobachtung von mindestens 2 Jahren und einem alpha-Fehler von 5 % wurde eine Aussagekraft von 80 % errechnet. Ein frühzeitiger Studienabbruch sollte in dem Fall erfolgen, wenn sich die reine Chemokonditionierung der TBI im Verlauf unterlegen zeigen sollte.

## Ergebnisse

Zwischen April 2013 und Dezember 2018 wurden 417 Patienten randomisiert, 212 in die TBI- und 201 in die Chemotherapiegruppe. Im März 2019 musste die Studie vorzeitig abgebrochen werden. Die mediane Nachbeobachtungszeit lag zu dieser Zeit bei 2,1 Jahren. Das Überleben nach zwei Jahren war in der TBI-Gruppe signifikant besser als in der Chemotherapiegruppe (91 % vs. 75 %; *p* < 0,0001). Die kumulative Inzidenz für ein Rezidiv und die therapieassoziierte Letalität nach TBI lagen bei 12 % bzw. 2 % und nach Chemokonditionierung bei 33 % bzw. 9 % (*p* < 0,0001 bzw. *p* = 0,269).

## Schlussfolgerung der Autoren

Aufgrund des signifikant besseren Gesamtüberlebens und einer niedrigeren Rezidivrate nach einer Konditionierung mit TBI und Etoposid im Vergleich zur reinen Chemokonditionierung wird erstere zukünftig bei Kindern mit HR-ALL empfohlen, die älter als 4 Jahre sind.

## Kommentar

Die Bestrahlung als hochwirksame Therapiekomponente in der Behandlung der akuten lymphatischen Leukämie konnte über die vergangenen Jahrzehnte zunehmend durch intensivierte Chemotherapieregimen verlassen werden. Dieses führte unter Erhalt guter rezidivfreier Überlebensraten zu einer signifikanten Reduktion strahlentherapieinduzierter Spätfolgen wie Wachstumsdefizienz, einer anhaltenden Reduktion der kognitiven Leistungsfähigkeit, Störungen der zentralen Hormonsteuerung, Ausbildung von Katarakten und Zweitmalignomen [1]. Busulfan mit seiner ausgezeichneten Liquorgängigkeit und seinen myeloablativen Eigenschaften wurde in der Transplantation bereits früh als Ersatz für eine Ganzkörperbestrahlung (TBI) eingeführt und deshalb auch gelegentlich als „liquid radiation“ bezeichnet. In der klassischen Kombination mit Cyclophosphamid war Busulfan aber mit nicht unerheblichen Akuttoxizitäten verbunden. In der Tat schien eine große Metaanalyse eine signifikant höhere therapieassoziierte Letalität nach busulfanbasierten Konditionierungsregimen zu belegen [2]. Auch für die TBI ist bzw. war der klassische Kombinationspartner Cyclophospahmid. Eine ursprünglich in Deutschland pilotisierte und später in die USA (City of Hope) überführte Kombination einer TBI mit Etoposid war mit vergleichbarem leukämiefreien Überleben und relativ guter Verträglichkeit assoziiert und deshalb Jahre später konzeptionell für die Pädiatrie übernommen und im Rahmen des Berlin-Frankfurt-Münster-Studienverbundes wieder nach Europa repatriiert worden.

Im ALL-SCT-BFM-2003-Trial wurde damit bei guter Leukämiekontrolle eine bis dahin unerreicht niedrige therapieassoziierte Letalität im einstelligen Prozentbereich erzielt [3]. Inzwischen waren Fludarabin und Thiotepa als relativ gut tolerierte und antileukämisch hochwirksame Agentien als Partner für Busulfan in reinen Chemokonditionierungen bei ALL verwendet worden. Treosulfan als dem Busulfan ähnliche, aber einfacher zu handhabende Substanz, verdrängte in einigen europäischen Ländern zunehmend das Busulfan in der HSCT [4]. Die sich nun aufdrängende wichtige Frage, ob diese wirksamen und relativ gut verträglichen Chemokonditionierungen in Zukunft eine TBI-haltige Konditionierung bei Kindern mit ALL ersetzen und damit entscheidend zur Verringerung strahlenassoziierter Nebenwirkungen nach HSCT beitragen könnten, muss nach dieser wichtigen randomisierten Studie wegen der signifikant schlechteren Leukämiekontrolle ohne TBI als negativ beantwortet werden.

## Fazit

Dies ist die erste randomisiert durchgeführte Studie, die prospektiv eine TBI-basierte vs. eine reine Chemokonditionierung (beide myeloablativ) hinsichtlich eines leukämiefreien Überlebens nach HSCT vergleicht. Bei Kindern und Jugendlichen mit ALL erzielt eine Konditionierung mit Ganzkörperbestrahlung (12 Gy, aufgeteilt auf 6 Fraktionen über 3 Tage) in Kombination mit Etoposid (1,8 g/m^2^; Höchstdosis: 3,6 g) eine signifikant bessere Leukämiekontrolle bei tendenziell besserer Akutverträglichkeit als eine Chemokonditionierung, bestehend aus Fludarabin (30 mg/m^2^ einmal täglich an 5 aufeinanderfolgenden Tagen), Thiotepa (5 mg/kg zweimal am Tag an einem Tag) und entweder Treosulfan (14 g/m^2^ einmal am Tag an 3 aufeinanderfolgenden Tagen) oder gewichtsadaptiertes Busulfan (an 4 aufeinanderfolgenden Tagen). Interessanterweise verlaufen dabei die Kurven für leukämiefreies Überleben mit dem gut liquorgängigen Busulfan im Vergleich mit dem wenig ZNS-gängigen Treosulfan fast identisch.

Diese Studie muss als Meilenstein in der hämatopoetischen Stammzelltransplantation angesehen werden und wird die Verwendung der Ganzkörperbestrahlung bei der pädiatrischen ALL auf Jahre hinaus als State of the Art festlegen.

*Martin G. Sauer, Hannover*
